# Weaning Age and Its Effect on the Development of the Swine Gut Microbiome and Resistome

**DOI:** 10.1128/mSystems.00682-21

**Published:** 2021-11-23

**Authors:** Devin B. Holman, Katherine E. Gzyl, Kathy T. Mou, Heather K. Allen

**Affiliations:** a Lacombe Research and Development Centre, Agriculture and Agri-Food Canada, Lacombe, Alberta, Canada; b USDA, ARS, National Animal Disease Center, Ames, Iowa, USA; Teagasc Food Research Centre

**Keywords:** swine, microbiome, metagenomics, resistome, weaning, CAZymes, antimicrobial resistance

## Abstract

Piglets are often weaned between 19 and 22 days of age in North America, although in some swine operations this may occur at 14 days or less. Piglets are abruptly separated from their sow at weaning and are quickly transitioned from sow’s milk to a plant-based diet. The effect of weaning age on the long-term development of the pig gut microbiome is largely unknown. Here, pigs were weaned at either 14, 21, or 28 days of age, and fecal samples were collected 20 times from day 4 (neonatal) through marketing at day 140. The fecal microbiome was characterized using 16S rRNA gene and shotgun metagenomic sequencing. The fecal microbiome of all piglets shifted significantly 3 to 7 days postweaning, with an increase in microbial diversity. Several *Prevotella* spp. increased in relative abundance immediately after weaning, as did butyrate-producing species such as Butyricicoccus porcorum, Faecalibacterium prausnitzii, and Megasphaera elsdenii. Within 7 days of weaning, the gut microbiome of pigs weaned at 21 and 28 days of age resembled that of pigs weaned at 14 days. Resistance genes to most antimicrobial classes decreased in relative abundance postweaning, with the exception of those conferring resistance to tetracyclines and macrolides-lincosamides-streptogramin B. The relative abundance of microbial carbohydrate-active enzymes (CAZymes) changed significantly in the postweaning period, with an enrichment of CAZymes involved in degradation of plant-derived polysaccharides. These results demonstrate that the pig gut microbiome tends change in a predictable manner postweaning and that weaning age has only a temporary effect on this microbiome.

**IMPORTANCE** Piglets are abruptly separated from their sow at weaning and are quickly transitioned from sow’s milk to a plant-based diet. This is the most important period in commercial swine production, yet the effect of weaning age on the long-term development of the pig gut microbiome is largely unknown. Metagenomic sequencing allows for a higher-resolution assessment of the pig gut microbiome and enables characterization of the resistome. Here, we used metagenomic sequencing to identify bacterial species that were enriched postweaning and therefore may provide targets for future manipulation studies. In addition, functional profiling of the microbiome indicated that many carbohydrate and metabolic enzymes decrease in relative abundance after weaning. This study also highlights the challenges faced in reducing antimicrobial resistance in pigs, as genes conferring tetracycline and macrolide resistance remained relatively stable from 7 days of age through to market weight at 140 days despite no exposure to antimicrobials.

## INTRODUCTION

In commercial swine production, the suckling-weaning transition is the most critical period for piglet health. When piglets are weaned, they are abruptly separated from their sow and their diet is changed from an easily digestible milk-based one to a more complex plant-based diet. The risk of developing health problems is increased as piglets are subjected to stress as a result of mixing with unfamiliar piglets, handling, and separation from the sow (1). This stress frequently leads to reduced feed intake immediately following weaning, which negatively affects growth performance (2). Consequently, newly weaned piglets frequently develop postweaning diarrhea, resulting in significant economic losses due to associated piglet morbidity, mortality, and treatment (3). Weaning times vary, but piglets can be weaned as young as 14 days or less in some North American commercial swine operations. Earlier weaning ages allow for a greater number of piglets weaned per sow per year and may also decrease the risk of transmission of certain pathogens from the sow to piglets. However, piglets that are weaned relatively early may be more susceptible to disease and other complications (4).

As with humans and other mammals, the gut microbiome is an important factor affecting swine health. There are an estimated 17 million plus microbial genes in the pig gut microbiome (5), compared to 20,000 to 25,000 genes in the swine genome (6). This greatly expands the genetic potential of the host, particularly as certain microbes can metabolize otherwise nondigestible dietary carbohydrates into a usable energy source. It has been well documented that the pig gut microbiome undergoes a rapid shift following weaning, including a decrease in members of the *Proteobacteria* phylum and *Bacteroides* genus and an increase in genera such as *Prevotella*, *Roseburia*, and *Succinivibrio* (7–10). However, relatively little is known about how weaning age affects the short- and long-term development of the pig gut microbiome. In this study, we weaned pigs at three different ages (14, 21, and 28 days) and collected fecal samples 20 times from the neonatal stage until they reached market weight. The fecal microbiome and resistome were assessed using 16S rRNA gene and shotgun metagenomic sequencing to determine how weaning age affected both over the course of the swine production cycle.

## RESULTS

### Effect of weaning age on pig performance.

**FIG 1:**
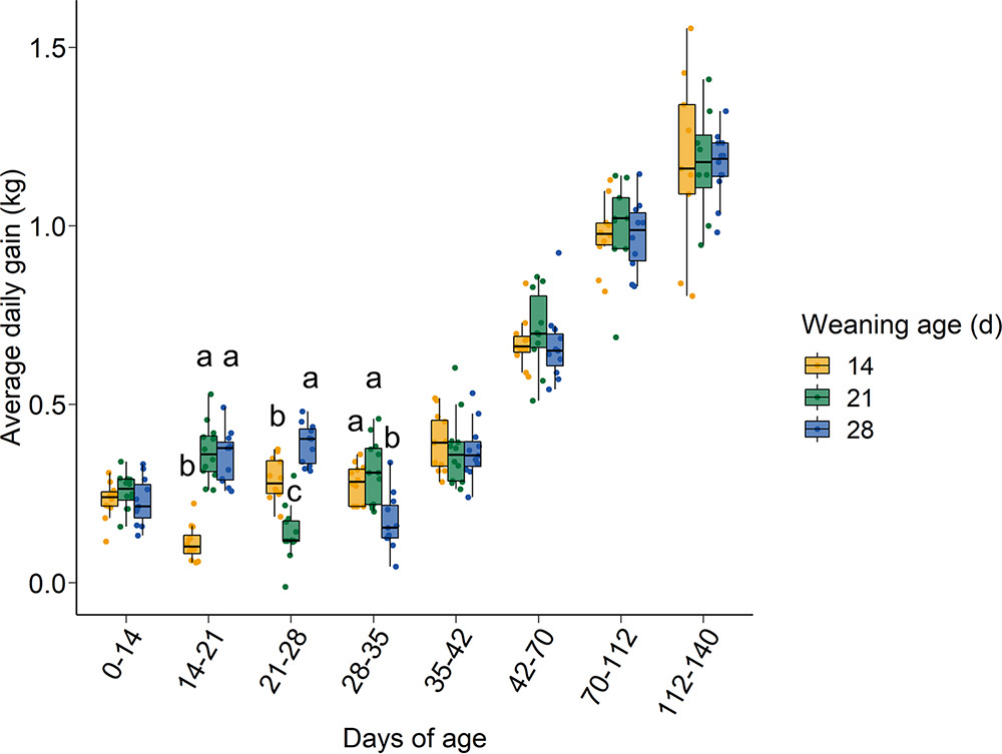
Average daily gain in kilograms of pigs by weaning age within each weighing period. Different lowercase letters indicate significantly different means (*P* < 0.05).

As expected, all pigs gained less weight in the 7-day postweaning period compared to pigs that were either still nursing or had already been on solid feed for longer than 7 days (Fig. 1). From day 35 onward, pigs from all weaning age groups grew at the same rate. There was also no association with weaning age and a pig being removed from the study due to antimicrobial treatment or death (*P* > 0.05).

### Sequencing.

The 16S rRNA gene sequencing of the mock community reflected the expected composition, with minor exceptions. There was a larger than expected relative abundance of *Clostridium* (see Table S1 in the supplemental material) and an absence of Cutibacterium acnes (formerly Propionibacterium acnes); however, this species is known to be poorly amplified by the primers used in this study (11). After processing, there were 35,448 ± 1,247 (mean ± standard error of the mean [SEM]) 16S rRNA gene sequences and 16,699,263 ± 680,292 shotgun metagenomic paired-end sequences per sample. For the metagenomic samples, host contamination accounted for 42.3% ± 2.0% of the sequences.

10.1128/mSystems.00682-21.3 TABLE S1 Percent relative abundance of genera identified in the mock communities (ATCC MSA-1002) via 16S rRNA gene sequencing. Note that *Schaalia* spp. are classified as *Actinomyces* spp. within the SILVA SSU database. Download Table S1, XLSX file, 0.01 MB. © Crown copyright 2021. 2021 Crown https://creativecommons.org/licenses/by/4.0/ This content is distributed under the terms of the Creative Commons Attribution 4.0 International license.

### Weaning age and the development of the gut microbiome.

**FIG 2:**
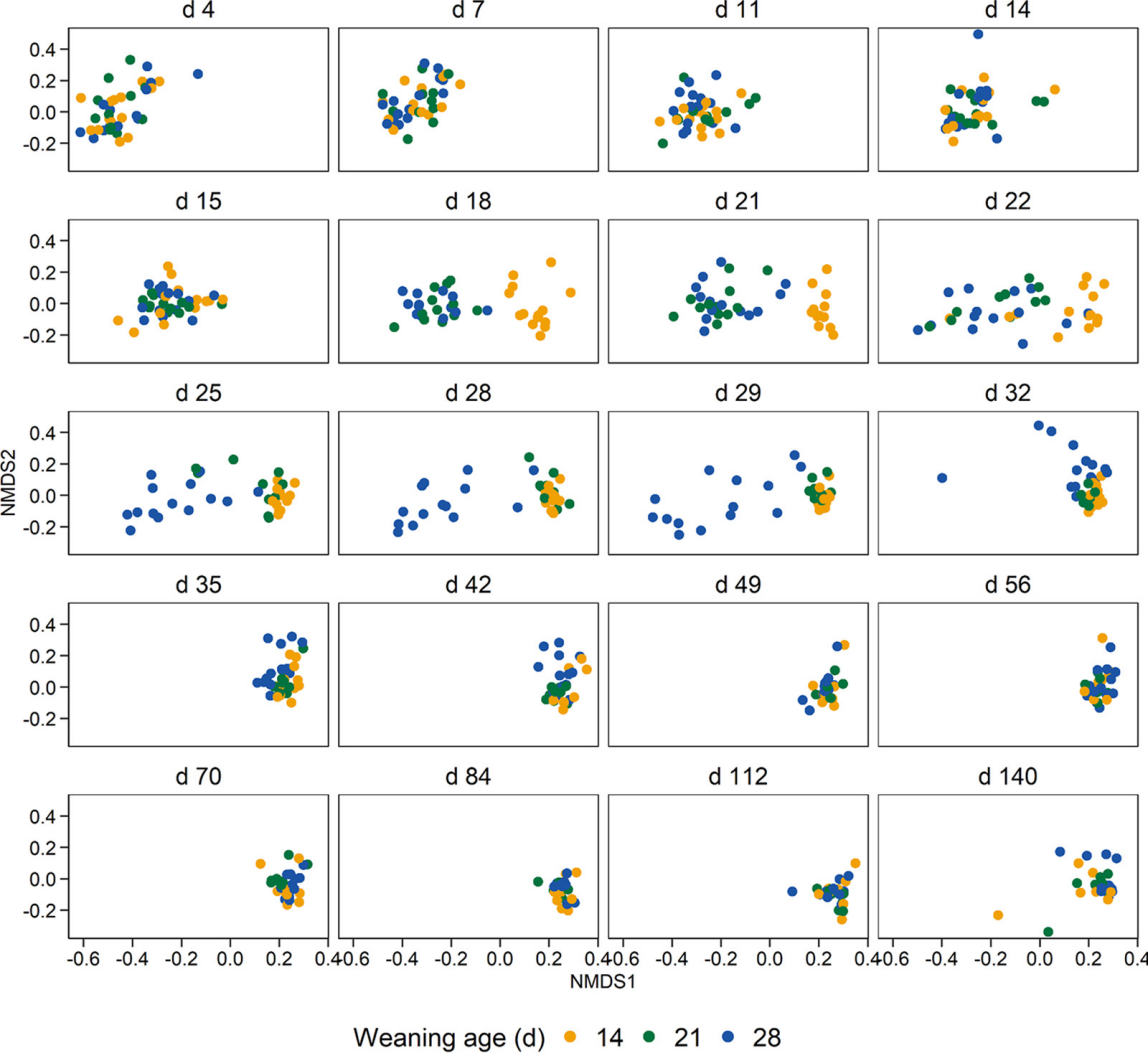
Nonmetric multidimensional scaling (NMDS2) plot of the Bray-Curtis dissimilarities for the pig fecal microbiota by weaning age and sampling day based on 16S rRNA gene sequencing.

Weaning age had a strong but temporary effect on the gut microbial community structure (Fig. 2; see Fig. S1 and S2 in the supplemental material). Within 3 days of weaning (day 18), the day-14-weaned pigs had a gut microbiota that was significantly different from that of the pigs that were still nursing (by permutational multivariate analysis of variance [PERMANOVA], *R*^2^ > 0.25 and *P* < 0.001). By 25 days of age, the gut microbiota of piglets weaned on day 21 was significantly different from that of both the day-14- and day-28-weaned groups (by PERMANOVA, *R*^2^ ≥ 0.13 and *P* < 0.001). However, on day 28, the day-14- and day-21-weaned piglets largely clustered together and separately from the day-28-weaned piglets, which were still nursing up to that point. Interestingly, the gut microbial community structure of piglets weaned at 28 days of age remained significantly different from that of the day-14-weaned pigs at day 35 and from the day-21-weaned pigs until and including day 42.

10.1128/mSystems.00682-21.1 FIG S1 Nonmetric multidimensional scaling plot of the Bray-Curtis dissimilarities for the fecal microbiota by weaning age and age of piglets. Download FIG S1, TIF file, 1.4 MB. © Crown copyright 2021. 2021 Crown https://creativecommons.org/licenses/by/4.0/ This content is distributed under the terms of the Creative Commons Attribution 4.0 International license.

10.1128/mSystems.00682-21.2 FIG S2 Frequency of fecal sampling of the pigs in this study. Download FIG S2, TIF file, 1.2 MB. © Crown copyright 2021. 2021 Crown https://creativecommons.org/licenses/by/4.0/ This content is distributed under the terms of the Creative Commons Attribution 4.0 International license.

**FIG 3:**
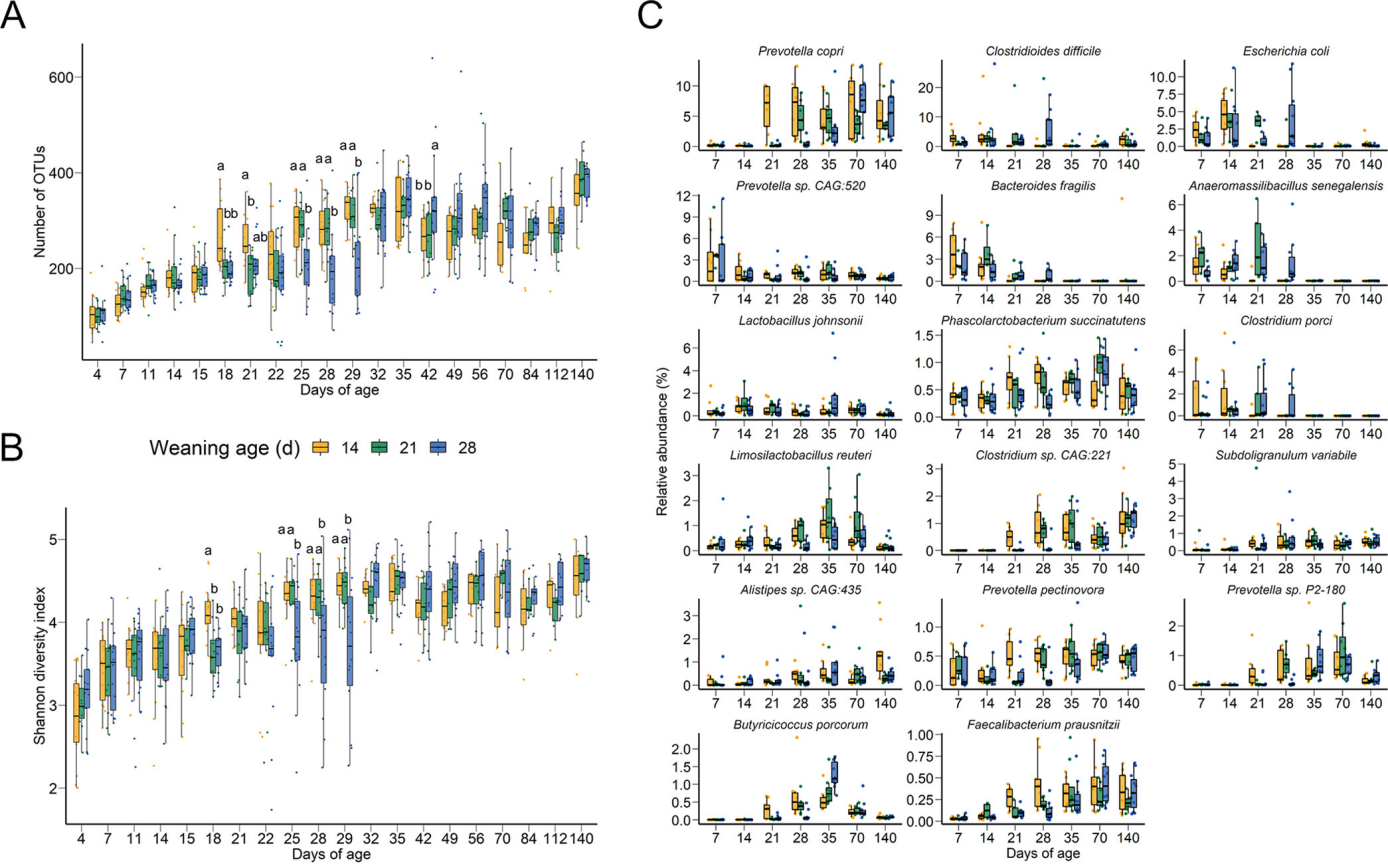
(A) Number of OTUs and (B) Shannon diversity index values based on 16S rRNA gene sequencing and (C) the 15 most relatively abundant bacterial species based on shotgun metagenomic sequencing for the pig fecal microbiome by weaning age and sampling day. In panels A and B, different lowercase letters indicate significantly different means (*P* < 0.05). In panel C, species are ordered by overall percentage of relative abundance. Butyricicoccus porcorum and Faecalibacterium prausnitzii are also included, based on their enrichment postweaning and butyrate-producing activities.

There was an increase in richness (number of operational taxonomic units [OTUs]) and diversity (Shannon diversity index) 4 days postweaning in the day-14-weaned piglets compared to the still-nursing piglets (Fig. 3A and B). Similarly, from days 25 to 29, both the day-14- and day-21-weaned piglets had greater diversity and richness than the still-nursing day-28-weaned group. These differences had disappeared by day 32, and with the exception of day 42, when the day-28-weaned piglets had a richer microbiota than the other two groups, the diversity of the piglet gut microbiota was not affected by age at weaning. Based on the shotgun metagenomic sequencing analysis, the shifts observed in the gut microbiome postweaning were associated with a number of different bacterial species (Fig. 3C; see Tables S3 and S4 in the supplemental material). Among those that increased in relative abundance postweaning were several *Prevotella* spp., including Prevotella copri, Prevotella pectinovora, *Prevotella* sp. strain P2-180, *Prevotella* sp. strain P3-122, and Prevotella stercorea. Butyricicoccus porcorum, Faecalibacterium prausnitzii, Selenomonas bovis, and Treponema porcinum were also among those significantly enriched in pigs that had been weaned at either day 14 or 21 compared to piglets that were not weaned until day 28 (*P* < 0.05).

Bacterial species that were consistently associated with nursing pigs included Anaeromassilibacillus senegalensis, Bacteroides fragilis, Clostridioides difficile, Clostridium porci, Clostridium scindens, Desulfovibrio piger, Escherichia coli, Phocaeicola vulgatus, and Shigella sonnei (see Table S4 in the supplemental material). At 35 days of age, only three bacterial species were differentially relatively abundant between the day-14- and day-21-weaned pigs and those weaned on day 28: Bariatricus massiliensis, *B. porcorum*, and *D. piger*, all of which were enriched in the day 14-weaned pigs (Table S4). Once the pigs had reached 70 days of age, there were no bacterial species with a relative abundance greater than 0.1% that differed among the groups (*P* > 0.05).

10.1128/mSystems.00682-21.4 TABLE S2 Pairwise PERMANOVA of the Bray-Curtis dissimilarities by weaning age within each sampling time. Download Table S2, XLSX file, 0.01 MB. © Crown copyright 2021. 2021 Crown https://creativecommons.org/licenses/by/4.0/ This content is distributed under the terms of the Creative Commons Attribution 4.0 International license.

10.1128/mSystems.00682-21.5 TABLE S3 Percent relative abundance (±SEM) of microbial species by weaning age and sampling time based on metagenomic sequencing. Only those microbial species with a relative abundance greater than 0.01% are included. Species are listed by overall percentage of relative abundance. Download Table S3, XLSX file, 0.1 MB. © Crown copyright 2021. 2021 Crown https://creativecommons.org/licenses/by/4.0/ This content is distributed under the terms of the Creative Commons Attribution 4.0 International license.

10.1128/mSystems.00682-21.6 TABLE S4 Differentially abundant microbial species based on metagenomic sequencing. Negative coefficient values (highlighted) at 21 days of age indicate that the microbial species was more relatively abundant in the day-14-weaned pigs versus the day-21- and day-28-weaned pigs. Negative coefficient values (not highlighted) at 28 and 35 days of age indicate that the microbial species was more relatively abundant in the day-28-weaned pigs versus the day-14- and day-21-weaned pigs. Download Table S4, XLSX file, 0.02 MB. © Crown copyright 2021. 2021 Crown https://creativecommons.org/licenses/by/4.0/ This content is distributed under the terms of the Creative Commons Attribution 4.0 International license.

### Functional changes in the microbiome postweaning.

**FIG 4:**
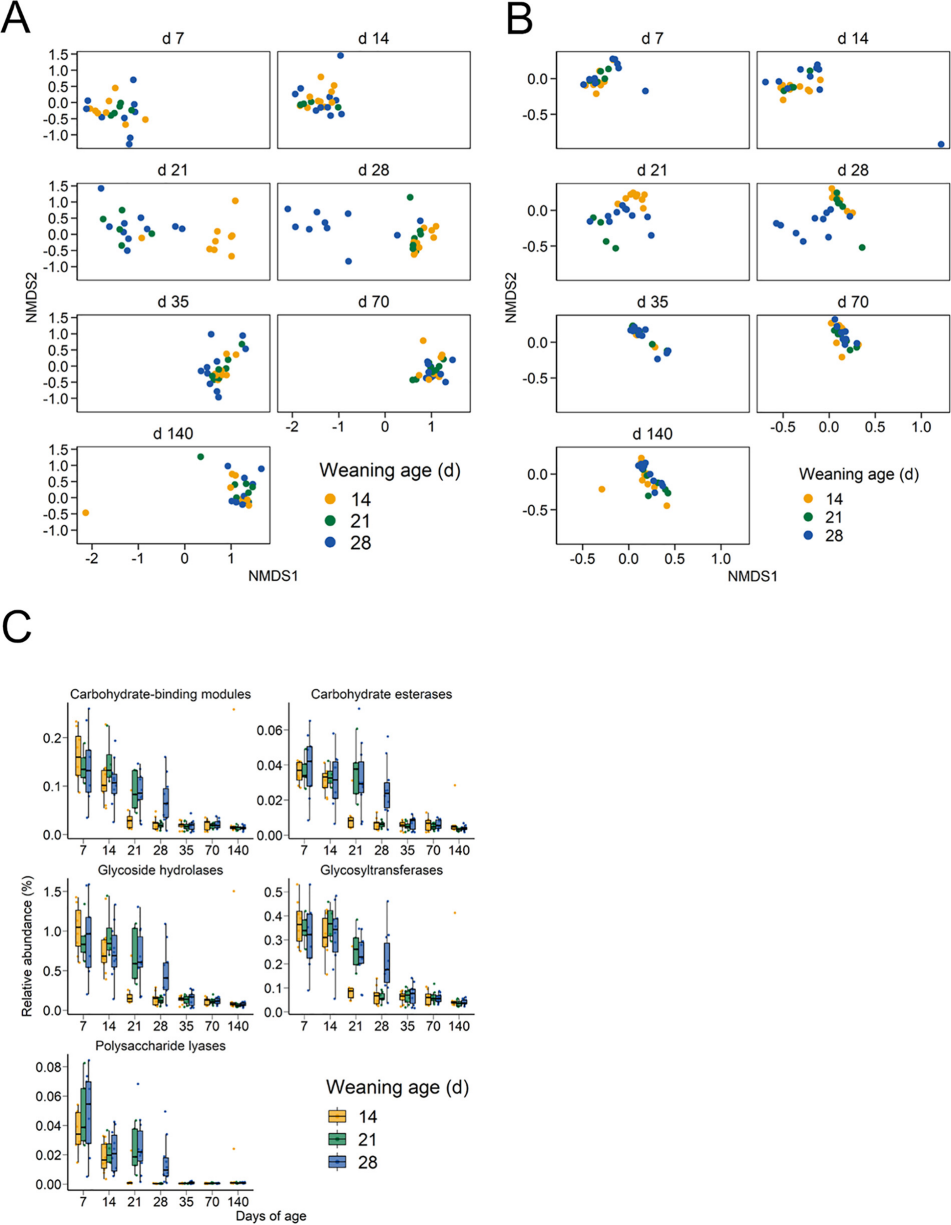
Nonmetric multidimensional scaling (NMDS) plot of the Bray-Curtis dissimilarities for the (A) CAZymes and (B) MetaCyc metabolic pathways of the pig fecal microbiome and (C) percentage of relative abundance of CAZyme classes by weaning age and sampling day.

Functional profiling of the gut microbiome was carried out using the MetaCyc metabolic pathway database and the CAZy database of carbohydrate-active enzymes (CAZymes). The relative abundance of the CAZymes and MetaCyc pathways shifted in a similar way to the microbial taxa postweaning (Fig. 4A and B). The CAZymes are grouped into the following classes: auxiliary activities (AAs), carbohydrate esterases (CEs), glycoside hydrolases (GHs), glycosyltransferases (GTs), polysaccharide lyases (PLs), and carbohydrate-binding modules (CBMs), which have no enzymatic activity but aid and enhance the catalytic activity of other CAZymes. In total, 237 CAZy families were detected among all samples (see Table S5 in the supplemental material), in comparison with only 61 found within the pig genome (see Table S6 in the supplemental material). All of the CAZyme classes decreased in relative abundance after weaning (Fig. 4C). Overall, 61.5% of the CAZymes were classified as glycoside hydrolases and 24.7% as glycosyltransferases. However, there were still a number of CAZy families that were enriched in the gut microbiomes of postweaned pigs compared to those still nursing (see Table S7 in the supplemental material). The only AA identified was AA10 (copper-dependent lytic polysaccharide monooxygenases) and in only 35 of the samples (Table S5).

10.1128/mSystems.00682-21.7 TABLE S5 Percent relative abundance (±SEM) of CAZy families detected in at least one sample by weaning age and sampling time. Download Table S5, XLSX file, 0.07 MB. © Crown copyright 2021. 2021 Crown https://creativecommons.org/licenses/by/4.0/ This content is distributed under the terms of the Creative Commons Attribution 4.0 International license.

10.1128/mSystems.00682-21.8 TABLE S6 CAZy families detected in the Sus scrofa genome at 90% identity. Download Table S6, XLSX file, 0.01 MB. © Crown copyright 2021. 2021 Crown https://creativecommons.org/licenses/by/4.0/ This content is distributed under the terms of the Creative Commons Attribution 4.0 International license.

10.1128/mSystems.00682-21.9 TABLE S7 Differentially abundant CAZy families on days 21 and 28 between weaned versus nursing piglets. Negative coefficient values (highlighted) at 21 days of age indicate that the CAZy family was more relatively abundant in the day-14-weaned pigs versus the day-21- and day-28-weaned pigs. Negative coefficient values (not highlighted) at 28 days of age indicate that the CAZy family was more relatively abundant in the day-28-weaned pigs versus the day-14- and day-21-weaned pigs. Download Table S7, XLSX file, 0.05 MB. © Crown copyright 2021. 2021 Crown https://creativecommons.org/licenses/by/4.0/ This content is distributed under the terms of the Creative Commons Attribution 4.0 International license.

At 21 days of age, there were 141 unique CAZy families that were differentially abundant between the day-14-weaned pigs and the day-21- and day-28-weaned piglets that were still nursing (*P* < 0.05) (Table S7). Similarly, at 28 days of age, 134 CAZy families were differentially abundant between the still nursing day-28-weaned piglets and the postweaned day-14- and day-21-weaned pigs (*P* < 0.05) (Table S7). There were no differences in CAZy family relative abundance among the three weaning age groups by day 35 (*P* > 0.05). Many of the alterations in the CAZyme profiles postweaning reflect the change in diet with CAZy families, with lactose degradation activity (GH2 and GH42) and activity against other components of porcine milk oligosaccharides (PMOs) (GH16, GH18, GH20, GH29, GH30, GH35, GH95, GH139, and GH141) enriched in pigs that were nursing compared to those that had been weaned. Meanwhile, CAZy families, including CBMs with mannan- pectin-, starch-, and xylan-binding functions (CBM23, CBM25 CBM26, and CBM77) and GHs with activity against plant cell carbohydrates (GH5, GH39, GH48, GH53, GH93, and GH94) (12, 13), were more relatively abundant in postweaned pigs that were consuming only a plant-based solid feed.

A large number of MetaCyc metabolic pathways were also differentially abundant between weaned and nursing piglets at day 21 (196 unique pathways) and day 28 (231 unique pathways), with the majority enriched in the gut microbiome of nursing piglets (see Table S8 in the supplemental material and Table S9 at https://doi.org/10.6084/m9.figshare.c.5619817.v1). As with the CAZymes there was an enrichment of MetaCyc pathways involved in fucose and lactose degradation in the nursing piglets and an increased relative abundance of certain starch degradation pathways postweaning.

10.1128/mSystems.00682-21.10 TABLE S8 Percent relative abundance (±SEM) of MetaCyc pathways detected in at least one metagenomic sample by weaning age and sampling time. Download Table S8, XLSX file, 0.1 MB. © Crown copyright 2021. 2021 Crown https://creativecommons.org/licenses/by/4.0/ This content is distributed under the terms of the Creative Commons Attribution 4.0 International license.

### Weaning age and the gut resistome.

**FIG 5:**
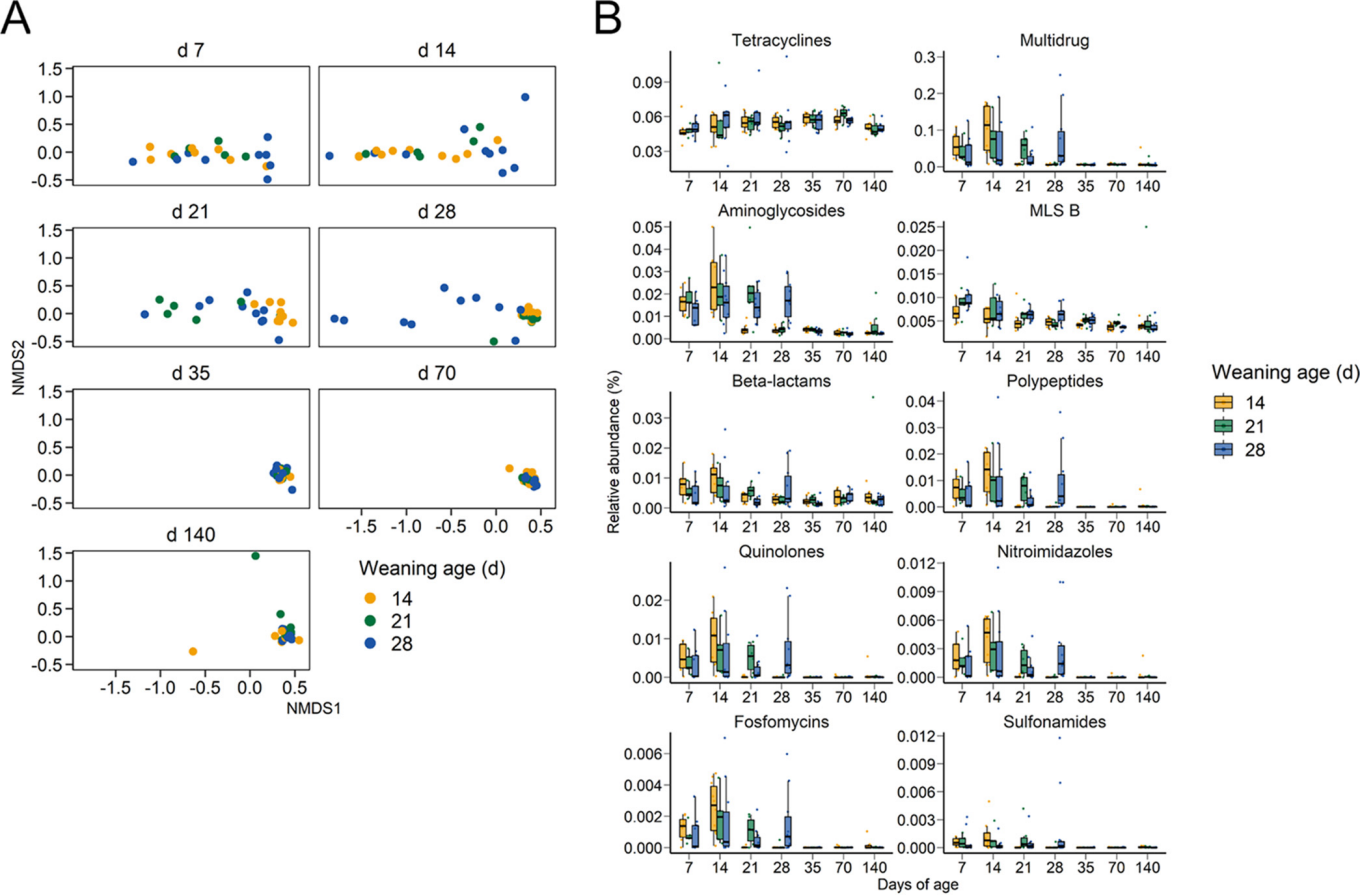
Nonmetric multidimensional scaling (NMDS) plot of the Bray-Curtis dissimilarities for the (A) antimicrobial resistance genes and (B) percentage of relative abundance of antimicrobial resistance genes by antimicrobial class by weaning age and sampling day.

Antimicrobial resistance remains a serious challenge to the swine industry, and therefore, we also characterized the antimicrobial resistome of the pigs longitudinally and in response to weaning age. Similar to the functional analysis, samples clustered by weaning age on days 21 and 28, when assessed using the relative abundance of antimicrobial resistance genes (ARGs) (Fig. 5A). The large majority of ARGs that were differentially abundant were enriched in the nursing piglets compared to the weaned pigs (see Table S10 at https://doi.org/10.6084/m9.figshare.c.5619817.v1). Notable ARGs that were more relatively abundant in the weaned pigs included *bla*_ACI-1_, *cfxA6*, *erm*(Q), *tet*(44), and *tet*(L). The relative abundance of ARGs conferring resistance to multiple drugs, aminoglycosides, polypeptides, and quinolones, as well as several other drug classes, decreased postweaning in all weaning age groups (Fig. 5B). However, tetracycline resistance genes remained relatively stable throughout the pig production cycle. Of the 250 unique ARGs detected, *tet*(Q), *tet*(W), *tet*(O), *aph(3′)-IIIa*, *mel*, *tet*(W/N/W), *tet*(40), and *tet*(44) were the most relatively abundant among all samples (see Table S11 at https://doi.org/10.6084/m9.figshare.c.5619817.v1).

## DISCUSSION

As expected, there was a substantial shift in the pig gut microbiome within 3 days of weaning. The sudden change from a milk-based diet to one that is plant based and less digestible by the pig is largely responsible for this shift immediately postweaning (1, 14). However, weaning age had no apparent long-term effects on the gut microbiome or the average daily gain of the pigs. A recent study by Massacci et al. (15) that also weaned pigs at different ages (14, 21, 28, and 42 days), with sampling up to 60 days of age, also reported no weaning age effect on the microbial community structure at 60 days. Therefore, it appears that a later weaning age only delays postweaning changes in the gut microbiome rather than affecting the assembly and stability of the microbial community.

Several short-chain fatty acid (SCFA)-producing bacterial species were prevalent among those that were more relatively abundant in pigs that had been weaned. These included Anaerovibrio slackiae (acetate and propionate), *B*. *porcorum* (butyrate), Coprococcus catus (butyrate and propionate), *F*. *prausnitzii* (butyrate), Megasphaera elsdenii (acetate, butyrate, and propionate), Phascolarctobacterium succinatutens (propionate), *P*. *copri* (acetate), Prevotella mizrahii (acetate), *P*. *pectinovora* (acetate), and S. bovis (acetate and propionate) (16–21). Short-chain fatty acid production occurs mostly in the lower gastrointestinal tract of pigs as a result of bacterial fermentation of undigested carbohydrates (22). Acetate, butyrate, and propionate have anti-inflammatory effects on the host (23) and provide up to 25% of daily energy requirements in pigs (24). Butyrate in particular is the primary energy source of colonocytes and regulates apoptosis (25).

Interestingly, *F*. *prausnitzii* has also been reported to be more relatively abundant at 60 days of age in pigs weaned at 21, 28, and 42 days versus 14 days (15) and in healthy pigs versus those with postweaning diarrhea (26). Butyricicoccus porcorum has been associated with higher feed efficiency, as have Treponema porcinum and Treponema succinifaciens, which were also more relatively abundant in weaned pigs here (27). In-feed supplementation with Butyricicoccus pullicaecorum has been shown to improve health and feed efficiency in broiler chickens (28), and *F*. *prausnitzii* reduced intestinal permeability and cytokine expression in a mouse colitis model (29). Therefore, *B*. *porcorum* and *F*. *prausnitzii*, as well as potentially other bacterial species that were more relatively abundant in weaned pigs, are attractive targets for microbiome manipulation and further study into their role in pig gut health.

The bacterial species that were enriched in the microbial communities of pigs that were still nursing at days 21 and 28 include several potentially pathogenic species, such as C. difficile, E. coli, S. sonnei, and Streptococcus suis. It is difficult to assess virulence of these species here; however, the presence of potentially pathogenic bacteria preweaning may be a risk factor for postweaning morbidity and mortality (30). Although many of the more relatively abundant bacterial species were differentially abundant pre- and postweaning, several remained relatively stable throughout the pig production cycle. In particular, Lactobacillus johnsonii, Mogibacterium kristiansenii, and Subdoligranulum variabile were not affected by weaning. Among these bacterial species, L. johnsonii is the best described and has been reported to improve sow reproductive performance (31) and average daily gain in piglets during the first 35 days of life (32) when delivered in feed. *S*. *variabile*, a butyrate-producing bacterial species, is the only member of its genus and has been previously reported to be a member of the “core microbiota” of the pig gastrointestinal tract (33). *M*. *kristiansenii* has only recently been described and was originally isolated from pig feces (18).

The functional profile of the gut microbiome also shifted after weaning in all weaning age groups, similar to that of the taxonomic profiles. This included a decrease in the relative abundance of all CAZy families postweaning. The CAZymes encoded by the pig genome are greatly outnumbered by those in the gut microbiome, thereby providing the host with an additional source of energy, as discussed earlier. Sow’s milk contains not only lactose but at least 119 PMOs (34), which are composed of the monosaccharides fucose, galactose, glucose, *N*-acetylglucosamine, *N*-acetylgalactosamine, and sialic acid bound to a lactose or *N*-acetyllactosamine core (35). These PMOs are generally resistant to host digestive enzymes in the small intestine and are instead fermented by the colonic microbiome into SCFAs (36, 37).

In humans, *Bifidobacterium* and *Bacteroides* spp., including B. fragilis and *P. vulgatus* (formerly Bacteroides vulgatus), have been shown to metabolize human milk oligosaccharides (38). Bacteroides fragilis, which was among the most relatively abundant species in nursing piglets here, carries a number of glycoside hydrolase family genes that facilitate breakdown of milk oligosaccharides (39). Metabolites from the degradation of sialylated bovine milk oligosaccharides by B. fragilis have also been shown to enhance the growth of E. coli *in vitro* (40). All of the GH families found in B. fragilis (i.e., GH2, GH16, GH18, GH20, GH29, GH33, and GH95) were enriched in the gut microbiomes of nursing piglets. Similarly, GH families and CBMs associated with degradation of plant polysaccharides were more relatively abundant in fecal samples from pigs that had been weaned and consuming a solid plant-based diet for at least 7 days.

The relative abundance of ARGs within several antimicrobial classes decreased postweaning. However, ARGs conferring resistance to the tetracycline and MLS_B_ classes remained relatively stable throughout the study, despite the fact that none of the pigs were exposed to any antimicrobials. Not surprisingly, these are the antimicrobial classes with the longest history of use in swine production and are still among the most frequently administered antimicrobials in North American pigs (41, 42). This background level of tetracycline and MLS_B_ resistance probably also explains why several studies have reported limited or only temporary effects on the pig gut microbiome following exposure to drugs of these antimicrobial classes (8, 43, 44). The reason for the significant decrease in other ARGs after weaning is likely due to the postweaning shift in bacterial taxa carrying these ARGs. For example, many of the relatively abundant multidrug ARGs, such as *mdtF*, *acrF*, *evgS*, *acrB*, *mdtO*, *mdtP*, and *cpxA*, are found in the majority of E. coli and S. sonnei genomes, and both of these species decreased in relative abundance postweaning. In contrast, relatively abundant tetracycline resistance genes, such as *tet*(Q), *tet*(W), and *tet*(O), have a much wider host range (45).

Two of the ARGs that were more relatively abundant in weaned piglets compared to those still nursing were the Ambler class A β-lactamase genes *bla*_CfxA6_ and *bla*_ACI-1_. Additionally, *bla*_CfxA2_ was enriched in piglets weaned at days 14 and 21 compared to those still nursing on day 28. Both bla_CfxA2_ and bla_CfxA6_ have been identified in several *Prevotella* spp. (46), which likely accounts for the postweaning enrichment of these ARGs. In *Prevotella* spp., the *bla*_CfxA_ genes have been shown to confer resistance to ampicillin but not cefmetazole (47). The *bla*_ACI-1_ gene may be associated with *M*. *elsdenii*, as has been demonstrated in human gut metagenomes (48). Overall, these results again demonstrate the challenges faced when it comes to reducing antimicrobial resistance in swine as none of the pigs in this study were exposed to antimicrobials.

In conclusion, this study shows that weaning age has little effect on the long-term development and composition of the pig gut microbiome and resistome. Instead, the pig gut microbiome tends to change in a rather predictable manner postweaning in a swine production environment. Many ARGs also persisted in the feces of the pigs throughout the study, likely reflecting the long history of use of certain antimicrobial classes in swine production. Several bacterial species with potential beneficial properties such as SCFA production were found to be enriched postweaning and are attractive targets for future microbiome manipulation and culture-based studies.

## MATERIALS AND METHODS

### Animals and experimental design.

All pig experiments were carried out at the swine unit of the Lacombe Research and Development Centre. Seven pregnant sows that farrowed within 24 h of each other were used in the study. A total of 45 piglets (*n* = 15 per weaning age group) were randomly selected for inclusion in the study based on litter, weight, and sex, with low-weight piglets excluded. Following weaning, all pigs were fed the same starter diet that was free of antibiotics, prebiotics, and probiotics (see Table S12 at https://doi.org/10.6084/m9.figshare.c.5619817.v1). Any pig that required an antibiotic treatment was removed from the study. Animals in this experiment were cared for in agreement with the Canadian Council for Animal Care (2009) guidelines. The Lacombe Research and Development Centre Animal Care Committee reviewed and approved all procedures and protocols involving animals.

On day 4 prior to sampling, 15 piglets were randomly chosen from among the 7 litters and designated to be weaned at 14, 21, or 28 days of age (see Fig. S2 in the supplemental material). Piglets were sampled using fecal swabs (FLOQSwabs; Copan, Murrieta, CA, USA) beginning at 4 days of age and repeated on days 7 and 11. At 14 days of age, piglets assigned to the day-14 weaning group were removed from their sow after sampling and transferred to a nursery room within the swine barn. Fecal sampling continued for all piglets at 15, 18, and 21 days of age. On day 21, piglets in the day-21-weaned group were removed from their sow and placed in a nursery room. Fecal samples were taken from all pigs on days 22, 25, and 28, and on day 28, the day-28-weaned piglets were weaned from their sow and placed in the nursery room. Piglets were then sampled on days 29, 32, 35, 42, 49, 56, 70, 84, 112, and 140. All fecal swabs were immediately placed on ice, transported to the laboratory, and stored at −80°C until DNA extraction.

### DNA extraction and 16S rRNA gene and shotgun metagenomic sequencing.

DNA was extracted from fecal material collected on FLOQSwabs with the QIAamp BiOstic bacteremia DNA kit (Qiagen, Mississauga, ON, Canada) as per the manufacturer’s instructions, with the following modifications. Sterile scissors were used to remove the swab, which was then placed into a PowerBead tube with MBL solution and agitated at 70°C and 400 rpm for 15 min. After heating, the tubes were shaken in a FastPrep-24 (MP Biomedicals, Solon, OH, USA) at 4.0 m/s for 45 s. Tubes were allowed to rest in the MP FastPrep-24 for 5 min. Using sterile forceps, swabs were removed from PowerBead tubes prior to pelleting debris at 10,000 × *g* for 2 min. All remaining steps were followed as per the manufacturer’s protocol.

Extracted bacterial DNA was loaded onto nine 96-well plates, and two wells on each plate included a positive control (MSA-1002, 20 Strain Even Mix Genomic Material; ATCC, Manassas, VA, USA) and negative control (water). Negative extraction controls were also included. DNA was quantified and analyzed using the Qubit dsDNA HS assay kit (Thermo Fisher Scientific, Waltham, MA, USA) and Agilent high-sensitivity D1000 ScreenTape system (Santa Clara, CA, USA). The V4 hypervariable region of the 16S rRNA gene was amplified as per Kozich et al. (49). To prepare each 16S rRNA gene library, 5 μl of each sample from three 96-well plates was pooled at a time. The pooled library was normalized to 0.4 nM and submitted to the Genomics Facility in the Infectious Bacterial Diseases Research Unit at USDA-ARS-NADC in Ames, IA for 250-bp paired-end sequencing on a MiSeq instrument (Illumina, San Diego, CA) using v2 chemistry.

DNA from days 7, 14, 21, 28, 35, 70, and 140 of all pigs that remained in the study through day 140 was also subjected to shotgun metagenomic sequencing. Metagenomic libraries were prepared using 700 ng of DNA and the TruSeq DNA PCR-free library prep kit (Illumina, Inc.) following the manufacturer’s recommended protocol. Briefly, DNA was fragmented to an average length of 400 bp with a Covaris LE220 instrument, end repaired, A-tailed, and indexed with TruSeq Illumina adapters. Libraries were then validated on a Fragment Analyzer system with a high-sensitivity NGS fragment kit (Agilent Technologies, Mississauga, ON, Canada) to check for size and quantified by quantitative PCR (qPCR) using the Kapa Library Quantification Illumina/ABI Prism kit protocol (KAPA Biosystems, Wilmington, MA, USA). Equimolar quantities of each library were then pooled and sequenced on the Illumina NovaSeq 6000 instrument with an SP flowcell (2 × 250 bp) following manufacturer’s instructions.

### 16S rRNA gene sequence analysis.

The 16S rRNA samples were processed using DADA2 v.1.14 (50) in R v.3.6.3. Briefly, the forward and reverse reads were trimmed to 200 and 210 bp, respectively, merged with a minimum overlap of 75 bp, and chimeras removed. The RDP naive Bayesian classifier (51) and the SILVA SSU database release 138 (52) were then used to assign taxonomy to each merged sequence, referred to here as operational taxonomic units (OTUs) with 100% similarity. OTUs that were classified as chloroplasts, mitochondria, or eukaryotic in origin and those that were identified in the extraction control samples at an equal or higher abundance than in the biological samples were removed prior to analyses. The number of OTUs, Shannon diversity index, inverse Simpson’s diversity index, and the Bray-Curtis dissimilarities were calculated in R v.4.0.0 using Phyloseq 1.32.0 (53) and vegan v.2.5-6 (54). To account for uneven sequencing depth, all samples were randomly subsampled to 6,900 sequences per sample prior to analyses.

### Metagenomic sequence analysis.

Metagenomic sequences were trimmed (quality score of <15 over a sliding window of 4 bp; minimum length of 50 bp) and sequencing adapters removed using Trimmomatic v.0.38 (55). Bowtie2 v.2.4.2-1 (56) was used to align host sequences to the Sus scrofa genome (Sscrofa11.1) for removal. Taxonomy was assigned to the filtered metagenomic sequences using Kaiju v.1.7.3 (57) and the NCBI nonredundant protein database (13 October 2020). For functional profiling of the metagenomic samples, HUMAnN v.3.0.0.alpha.1 (58) was used to align reads to the UniRef90 database, which were then collapsed into MetaCyc metabolic and enzyme pathways (59). Reads were aligned to the Comprehensive Antibiotic Resistance Database (CARD) v.3.0.8 (60) and the Carbohydrate-Active enZYmes (CAZy) Database (dbCAN2) v.07312020 (61) using DIAMOND v.0.9.28 (62) (≥90% amino acid identity and ≥ 90% coverage).

### Statistical analysis.

Fisher’s exact test was used to determine if weaning age was associated with removal from the study postweaning due to antimicrobial treatment or death. The effect of weaning age on the microbial community structure was assessed using the Bray-Curtis dissimilarities and PERMANOVA (adonis2 function). The R package pairwiseAdonis (63) was used to compare the Bray-Curtis dissimilarities within each sampling time, and the Benjamini-Hochberg procedure was used to correct *P* values for multiple comparisons. The effect of weaning age on the relative abundance of microbial species, CAZy families, MetaCyc pathways, and ARGs was determined using MaAsLin2 (microbiome multivariable associations with linear models) v.1.5.1 (64) in R. Only those microbial species with an average relative abundance of at least 0.1% and CAZy families, MetaCyc pathways, and ARGs identified in at least 25% of samples were included in these analyses.

### Data availability.

All 16S rRNA gene and metagenomic sequencing data are available at the NCBI Sequence Read Archive under BioProject no. PRJNA629856.
