# Supplementary figures and images for: Weaning Age and Its Effect on the Development of the Swine Gut Microbiome and Resistome
Source: mSystems. 2021 Nov 23;6(6):e00682-21. doi: 10.1128/mSystems.00682-21 (PMC8609972; doi:10.1128/mSystems.00682-21)

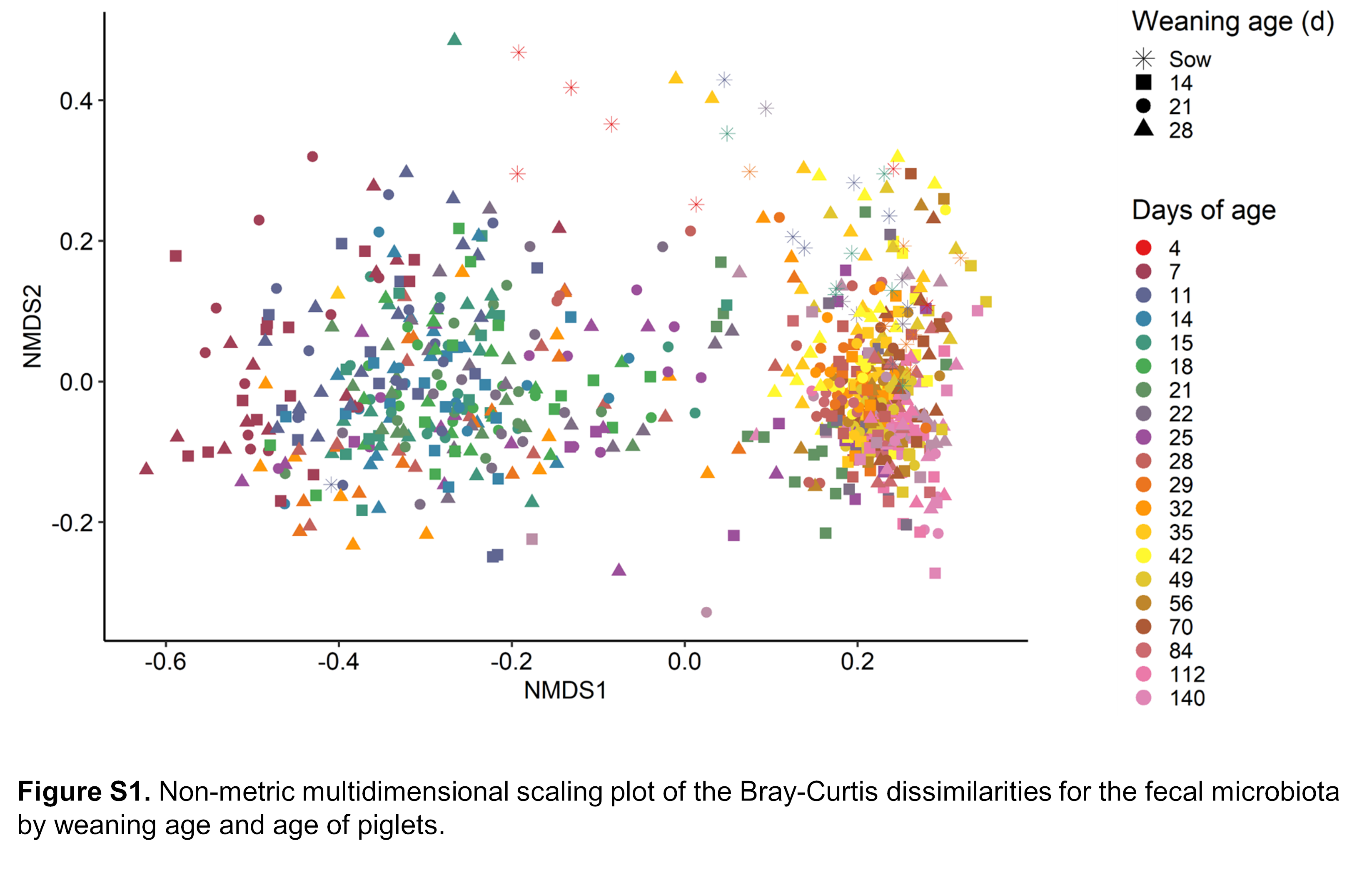

Supplement: FIG S1 [file msystems.00682-21-s0001.tif]

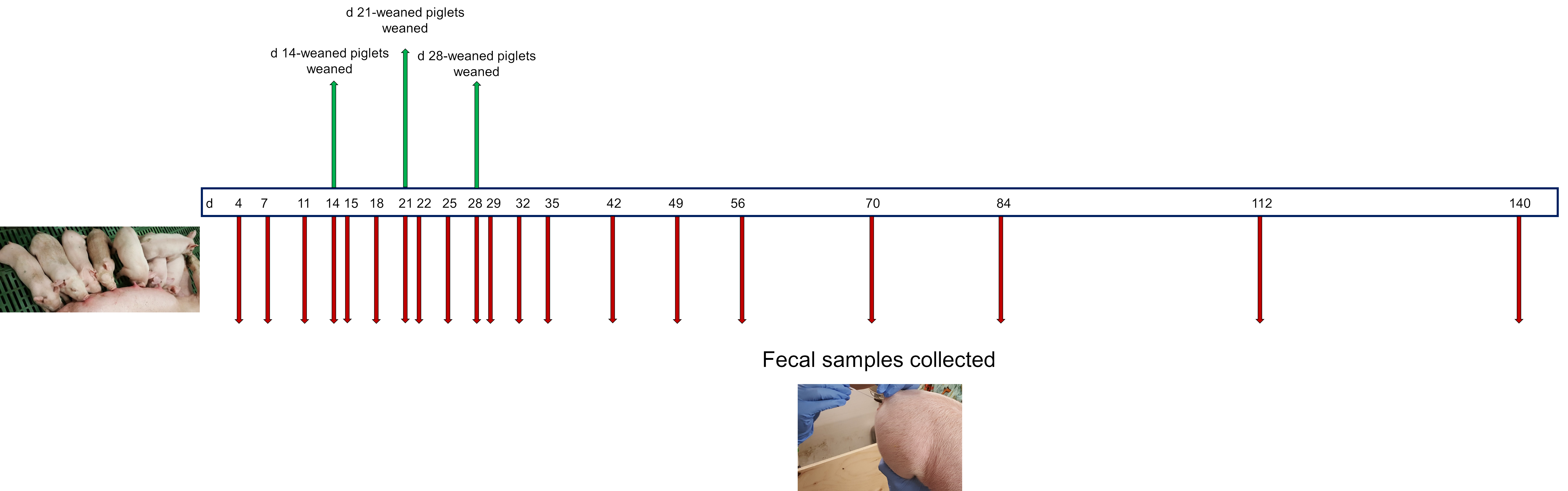

Supplement: FIG S2 [file msystems.00682-21-s0002.tif]
